# Retrospective evaluation of radiological and clinical outcomes after surgical treatment of proximal femoral fractures utilizing PFNA and PFNA augmented

**DOI:** 10.1007/s00402-024-05349-8

**Published:** 2024-05-03

**Authors:** Friedemann Schneider, Cedric Oettle, Armin Runer, Moritz Wagner, Rohit Arora, Richard A. Lindtner

**Affiliations:** 1 Department of Orthopaedics and Traumatology, Medical University of Innsbruck, Innsbruck, Austria; 2 Medical University of Innsbruck, Innsbruck, Austria; 3 Department for Orthopaedic Sports Medicine, Klinikum rechts der Isar, Technical University of Munich, Munich, Germany; 4 Department of Orthopaedics and Traumatology, Bezirkskrankenhaus St. Johann in Tirol, St. Johann in Tirol, Austria

**Keywords:** Femur, Proximal femoral fracture, Pertrochanteric fracture, Intertrochanteric fracture, Hip fracture, Proximal femoral nail, Cephalomedullary nail, Cement augmentation, PFNA

## Abstract

**Introduction:**

The primary aim of this study was to evaluate the clinical and radiological outcomes after surgical treatment of proximal femoral fractures utilizing the Proximal Femoral Nail Antirotation (PFNA), with the main focus on complications and reoperations. The secondary aim was to compare the outcomes of patients with and without cement augmentation of the cephalomedullary nails.

**Materials and methods:**

All patients with an acute proximal femoral fracture consequently treated with a PFNA between January 2011 and Dezember  2018 were evaluated. Clinical and radiological data were assessed for intra- and postoperative complications, including treatment failure. In addition, intra- and postoperative radiographs were used to determine the position of the implant, and any migration, via Tip-Apex-Distance (TAD) and the caput-collum-diaphyseal angle (CCD). The accuracy of the fracture reduction was rated according to Baumgaertners criteria.

**Results:**

Two hundred sixty-four consecutive patients (mean age 78.8 ± 12.0; 73.1% female) were included. The predominant OTA/AO fracture classification was 31A1 (153 cases, 58.0%). The average duration of surgery was 63.1 ± 28.0 min and showed no significant differences between PFNA and PFNA with augmentation. The implant positioning was rated as good in 222 cases (84.1%). Two hundred sixty-three patients (99.6%) showed evidence of healing within the time frame of three months postoperatively, one case of delayed union healed after secondary dynamization. During the observational period, 18 patients (6.8%) required a total of 23 additional surgeries. Overall, a lower reoperation rate was observed following the use of the augmentation option (2/86 patients (2.3%) vs. 16/178 patients (9.0%), *p* = 0.04). In particular, there were no cases of cut-out or cut-through among patients who underwent augmentation as part of osteosynthesis.

**Conclusions:**

Overall reoperation rate after surgical treatment of proximal femoral fractures utilizing the Proximal Femoral Nail Antirotation (PFNA) was 6.8%, with 23 additional surgeries performed in 18 patients. The usage of the PFNA with augmentation showed equally good implant positioning, excellent healing rates and fewer postoperative complications compared to the PFNA implant alone with a similar overall duration of surgery.

## Introduction

With increasing prevalence of osteoporosis and falls in a generally ageing population, there has also been an increase in proximal femur fractures. A further spread is to be expected as the number of elderly people is expected to continue to rise in the coming years, necessitating optimization of surgical treatment [1, 2]. Moreover, the rising incidence of proximal femur fractures results in increasing costs. Healthcare systems worldwide are confronted with these challenges. In addition, short- and long-term mortality increases after suffering a proximal hip fracture. Postoperative complications in particular have a major influence on morbidity and mortality [3].

Currently most trochanteric fractures are treated surgically, and intramedullary (IM) nailing with implants like the Proximal Femoral Nail Antirotation (PFNA, DePuy Synthes, West Chester, PA) offer fast and minimally invasive rotational and angular stability, which enables early mobilisation of the patient [4]. Yoo et al. [5] suggested increased body-mass index (BMI) and nonanatomic fracture reduction as risk factors for fixation failure after osteosynthesis using IM nailing in elderly patients with trochanteric fractures. The loss of microarchitecture and decrease in bone density in patients with osteoporosis results in an enlarged risk of harmful low-energy trauma. Especially for these patients the cement augmentation has been developed.

The cement is intended to provide an increased bone-implant contact surface. Therefore, improved anchoring can be achieved. The PFNA implant offers this option of controlled placement of cement around the implant, through the perforated blade with an injection cannula. This additional stability could be an advantage in terms of preventing complications such as “cut out” or “cut through” [6]. Cement-associated complications such as leakage of cement into the hip joint are rare and fractures treated with an augmented PFNA or the similar TFNAdvanced™ Proximal Femoral Nailing system (TFNA¸ DePuy Synthes, West Chester, PA) showed good levels of fracture healing [6–8].

The primary aim of this study was to evaluate the safety and performance of surgical treatment of proximal femoral fractures utilizing the Proximal Femoral Nail Antirotation (PFNA), with the main focus on complications and reoperations. The secondary aim was to compare the outcomes of patients with and without cement augmentation of the cephalomedullary nails. We hypothesised that patients treated with PFNA augmentation have less postoperative complications and revision operations compared to those without augmentation.

## Materials and methods

This study was conducted as a retrospective data evaluation consecutively including patients with a traumatic proximal femoral fracture that were treated with a PFNA with or without cement augmentation between January 2011 and December 2018. Augmentation with PMMA cement (Traumacem V+, DePuy Synthes, West Chester, PA) was considered based on patients´ age, other factors such as intraoperatively perceived bone quality and fracture pattern. The final decision was made by the surgeon in charge during the procedure.

The methodology of the present study is based on a previously published study by the authors [8]. In short, data was assessed by evaluating the patient chart documentation as well as the intra- and postoperative radiographs. No additional radiographs or examinations were performed. After verifying the inclusion and exclusion criteria, 264 patients were included, among them 186 patients with PFNA without augmentation and 86 patients treated surgically with PFNA augmentation.

Inclusion and exclusion criteria are presented in Table 1.

**Table 1:** Inclusion and exclusion criteria

Inclusion criteria	Exclusion criteria
● Age: ≥18–100 years ● All patients with acute traumatic fractures treated using the Proximal Femoral Nail Antirotation (PFNA) or the augmented PFNA ● AO Classification: pertrochanteric femoral fracture (31-A1 and 31-A2), trochanteric fracture (31-A3), fracture of the trochanteric area (31-A1/A2/A3) with diaphyseal extension or combined fracture of the trochanteric area and the femoral shaft (32-A/B/C) ● Intraoperative and immediate postoperative radiographs ● Minimum radiological follow-up of three months	● Age: <18 years ● Treatment using other than PFNA/PFNA augmented implant ● Non-traumatic fractures (E.g. metastatic fractures) ● Missing surgery protocol

The study was conducted in accordance with the Declaration of Helsinki. The primary objective of this study was to evaluate the intraoperative and postoperative implant-related complication rates.

Eight categories of implant-related complications were established, and every case was assigned to one of the following: (1) implant cut-out, (2) implant cut-through, (3) lateral protrusion of the screw or blade with pain, (4) medial migration of the shaft, (5) protrusion including distal anterior cortex, (6) implant breakage, (7) surgical site infection (SSI), (8) other implant-related complications requiring revision surgery.

Furthermore, additional surgeries were evaluated. A Charlson Comorbidity Index (CCI) score was calculated as described by Charlson et al. [9, 10]. Mortality was determined by querying the public provincial register. Patient-related data, such as alcohol intake, smoking status, preexisting diagnosis of osteoporosis, dementia and neurological disorders, were obtained by retrospective medical chart review.

The immediate postoperative radiographs in anterior-posterior (ap) and lateral (lat) view were used to determine the position of the blade/screw in the femoral head according to the work by Cleveland et al. [11], which results in nine possible positions (center–center, center–anterior, center–posterior, inferior–anterior, inferior–center, inferior–posterior, superior–anterior, superior–center, superior–posterior).

The tip-apex-distance (TAD) was calculated as described by Baumgaertner et al. [12]. The position of the nail was according to Schmutz et al. [13], divided into five different positions using the lateral radiographs (far anterior, anterior, central, posterior, far posterior).

To determine the extent of lateral migration of the blade/screw-head element, the length of the blade/screw protruding laterally from the intramedullary nail was measured in ap and lat views in all control radiographs performed.

Fracture reduction was rated according to Baumgaertner’s criteria [12] as “good,” “acceptable” or “poor” with normal or slight valgus alignment on the ap X-ray images, less than 20°angulation on the lateral view X-ray images, and less than 4 mm displacement for any fragment. If no agreement was achieved, a third experienced surgeon was asked to find a decision.

“Cut-out” was defined as the cranial penetration of the PFNA blade or screw through the femoral head, “Cut-through” as the central penetration of the blade or screw into the joint and occasionally even into the small pelvis without varus collapse.

“Lateral protrusion” was defined as lateral migration of the spiral blade/screw-element commonly resulting in pain or discomfort due to irritation of the iliotibial band and lateral soft tissues.

All data were analysed using SPSS v.26 (IBM Corp.). For continuous and normal distributed data, the Student’s T test was applied to determine differences between both groups. For ordinal or non-normally distributed data, the Mann–Whitney U test was used. Related pre- and postoperative data were compared using the Wilcoxon signed-rank test. A Pearson Chi-square test was performed to compare dichotomous variables. The significance level was set to 0.05 (two-sided) with 95% confidence intervals (CI).

## Results

Within the observation period a total of 264 consecutive patients were included (mean age 78.8 ± 12.0 years; range 31–99 years; 73.1% female). The mean Charlson Comorbidity Index (CCI) score was 6.16 ± 2.6. The average BMI was 24.3 ± 4.4. According to the medical records, almost two thirds (66.3%) of the patients were prediagnosed with osteoporosis at the time of surgery. Forty-three patients (16.3%) had a prior diagnosis of dementia at the time of surgery. A total of 45 patients (17.0%) reported regular alcohol consumption (at least once a week). Twenty-five patients (9.5%) stated that they were affected by an underlying neurological disease.

A detailed list of the reported risk factors is presented in Table 2.

**Table 2:** Prevalence of risk factors in the cohort

Risk factors	Number of patients (*n*)		Percentage (%)
Alcohol Smoking Osteoporosis Dementia Neurological disorder		45 43 175 43 25	17.0 16.3 66.3 16.3 9.5

The fractures were classified using the corresponding AO classification, with the simple pertrochanteric fracture (31A1) occurring most frequently (*n* = 153, 58.0%). In 86 patients (32.6%), an additional cement augmentation was performed. The average duration of surgery was 63.1 ± 28.0 min (min). No significant differences in the mean duration of surgery could be found between the augmented (64.0 ± 25.3 min) and the not-augmented (62.8 ± 29.2 min) PFNA-treatment. The implant positioning was rated as good in 222 cases (84.1%) with 93.2% showing a center-center position of the blade/screw in the femoral head. The mean TAD was 21.5 mm.

Table 3 presents the frequencies of the relevant classification categories.

**Table 3:** Frequencies of the relevant AO classification categories

AO Fracture Classification	Number of patients (*n*)	Percentage (%)
31A1	153	58
31A1 + 32C	8	3
31A2	70	26.5
31A2 + 32C	4	1.5
31A3	11	4.2
31B	11	4.2
32A	7	2.6
Total	264	100

During the observation period, 23 additional surgeries were performed on 18 patients (6.8%). Patients with postoperative complications were significantly younger than those without postoperative complications (70.8 ± 12.4 vs. 79.3 ± 11.8 years, *p* = 0.01). Similarly, there were significant group differences (*p* = 0.008) in the mean CCI score, which was 4.6 ± 2.4 in the group with postoperative complications and 6.3 ± 2.6 in the group without.

Patients with augmentation showed a significantly lower rate of postoperative complications (*p* = 0.04). Only two of the 86 patients treated with PFNA augmentation underwent additional surgeries (2.3%), including one exchange of the blade after symptomatic lateral blade protrusion and two haematoma evacuations in one patient. In particular, no cases of cut-out or cut-through were observed after augmentation. In the PFNA without augmentation group, 16 patients out of 178 had postoperative complications necessitating additional surgeries (9%). Three patients required a total of five hematoma removals. Four blades had to be exchanged due to persistent pain at the lateral end of the blade. One case of mal-rotation was treated surgically. Furthermore, 11 patients required a total of 12 implant removals, including two (0.8%) blade removals, one (0.4%) partial implant removal of a distal screw and nine (3.4%) complete implant removals, three with consequent joint replacement (details see Table 4). Sub-group analysis of the 175 patients with a preexisting diagnosis of osteoporosis revealed a lower complication and reoperation rate (2/64 (3.1%) vs. 7/111 (6.3%) for each comparison) in the PFNA with augmentation group. However, these differences did not reach significance (*p* = 0.36 for each comparison).

Table 4 gives an overview of all patients with postoperative complications as well as the respective treatments performed.

Overall, two hundred sixty-three patients (99.6%) showed evidence of healing (callus formation / bone union) within the time frame of three months postoperatively, one case of delayed union healed after secondary dynamization.

It must be noted that the number of patients attending the respective check-up appointments varied considerably over the course of the study. All 264 patients were observed at the first postoperative control after 5–14 days. After two to three months, 251 patients (95.1%) returned for a follow-up. After 1-year the number of patients shrank further to 125 patients (47.3%).

Seven patients (2.7%) died within the first 12 months postoperatively, none within the first three months.

**Table 4:** Postoperative complications and consequent treatment

Pat. Nr.	Complication	Treatment	Additional surgeries	Augmented (yes = 1; no = 0)
1	Malrotation **+** Persistent discomfort / pain	De-Rotation Removal distal screw	2	0
2	Postoperative Haematoma	Haematoma evacuation	1	0
3	Postoperative Haematoma	2x Haematoma evacuation	2	0
4	Postoperative Haematoma	2x Haematoma evacuation	2	1
5	Delayed Union	Secondary dynamization	1	0
6	Deep infection	Implant removal	1	0
7	Lateral protrusions of the blade **+** Persistent discomfort / pain	Removal of the blade + subsequent complete Implant removal	2	0
8	Lateral protrusions of the blade	Removal of the blade	1	1
9	Lateral protrusions of the blade Cut through	Exchange of the blade + subsequent implant removal and prosthesis	2	0
10	Lateral protrusions of the blade	Exchange of the blade	1	0
11	Lateral protrusions of the blade	Exchange of the blade	1	0
12	Lateral protrusions of the blade	Exchange of the blade	1	0
13	Refracture after fall	Removal and prosthesis	1	0
14	Persistent discomfort / pain	Implant removal	1	0
15	Persistent discomfort / pain	Implant removal	1	0
16	Persistent discomfort / pain	Implant removal	1	0
17	Persistent discomfort / pain	Implant removal	1	0
18	Cut out	Removal and prosthesis	1	0
		9 Complete removals 3 Partial removals 4 Exchange of blades 1 Secondary dynamization 5 Haematoma evacuations 1 Derotation	**23**	**2**

## Discussion

We evaluated the clinical and radiologic outcome after surgical treatment of proximal femoral fractures utilizing the Proximal Femoral Nail Antirotation (PFNA). Overall reoperation rate was 6.8%, with 23 additional surgeries performed in 18 patients. Patients requiring revision surgery were significantly younger (70.8 ± 12.4 vs. 79.3 ± 11.8 years, *p* = 0.01) and CCI score was lower (4.6 ± 2.4 vs. 6.3 ± 2.6, *p* = 0.008). The PFNA with cement augmentation group showed a significantly lower reoperation rate than that without augmentation (2/86 patients (2.3%) vs. 16/178 patients (9.0%), *p* = 0.04). In particular, no cases of cut-out or cut-through were observed after augmentation.

The clinical evidence that augmentation of the PFNA blade reduces complications of proximal femoral fracture fixation is limited so far. However, in this study we found a significantly lower overall complication rate and no case of cut out/cut through in the augmentation group. In view of the fact that augmentation was particularly applied in cases of poor bone quality and/or in unstable fracture patterns as repeatedly documented in the surgery protocols, this seems to confirm the beneficial effect of augmentation on the mechanical stability of proximal femoral fracture fixation. A recent meta-analysis by Rompen et al. [14] assessing a variety of different implants (including different cephalomedullary nails and dynamic hip screws) similarly concluded that cement augmentation for trochanteric femur fractures leads to fewer complications, re-operations and shorter hospital stays at the expense of a slightly longer operation duration (7 min on average). Significantly longer operation times for additional augmentation were reported by Böhringer et al. [15] with significantly higher costs, taking into account the price of the augmentation kit and the additional operation time. The authors raised the question whether a more expensive cement augmentation might avoid more costly revision surgery and would justify the costs. Two studies evaluating the socioeconomic impact and cost effectiveness of cement augmentation for the fixation of unstable trochanteric fractures found that augmentation was associated with cost savings [16, 17]. Moreover and in contrast to Böhringer et al. [15], the mean operation time was only slightly higher for the augmented PFNA in the present study (64.0 vs. 62.8 min). For both treatments, the average surgery duration is in line with further pre-existing literature [6, 18]. Obviously, the augmentation option could not prevent non-implant-related reasons for additional surgery such as hematoma removals. Considering the average age of the study participants and the general frequency of therapeutic anticoagulation in this age category, side effects such as postoperative haematoma are to be expected independently of the augmentation option given the size of the observed study cohort [1, 3, 6].

The calculated mean CCI score of 6.16 ± 2.6 corresponds to an estimated 10-year survival rate of around 2% [9, 10]. Considering this calculated mortality rate, the actual observed all-cause mortality of 2.7% within the first 12 months postoperatively appears low. The PFNA can therefore be considered safe in terms of mortality and observed complications. The mean age in this study was 78.8 years and included only a few young patients. More precisely, three high-energy trauma patients aged 31, 41 and 44 years were included. On the one hand these three patients reduced the average age of the study cohort to some extent, on the other hand the mean age of patients with postoperative complications was 70.8 years and did not include any of these high-energy trauma patients. Similar to the authors’ previous study [8], the present study also showed a lower mean age and CCI score for patients with postoperative complications and re-operations, for which several explanations may exist. First, assuming a more active lifestyle of a younger and healthier collective, the daily load on the operated body region could be higher. Second, from a surgical point of view, the surgeons may have been more cautious in indicating re-operation for mild complaints in the older population with more comorbidities.

The study limitations include the retrospective study design with a decrease in the follow-up rate with increasing time from surgery. The latter is a well-known issue of studies on geriatric patients and may result in an underestimation of the complication rate. However, as our institution represents the largest public hospital in the region and serves as a tertiary referral center, it is unlikely that patients who had experienced a complication requiring treatment have been admitted to a hospital other than ours. Moreover, osteoporosis may be underdiagnosed in our cohort as no systematic bone densitometry measurements were available due to the retrospective study design. Finally, the high proportion of women in the study population may limit the generalizability of the results to the male population. Nevertheless, the mean age of the cohort and the high proportion of women is in line with the pre-existing literature on proximal femur fractures and can be assumed to be typical for the injury pattern investigated [1, 6].

## Conclusion

Overall reoperation rate after surgical treatment of proximal femoral fractures utilizing the Proximal Femoral Nail Antirotation (PFNA) was 6.8%, with 23 additional surgeries performed in 18 patients. The usage of the PFNA with augmentation showed equally good implant positioning, excellent healing rates and a lower reoperation rate compared to the PFNA implant alone with a similar overall duration of surgery.
